# *Predictive Value of HE4 in Platinum-Based Chemotherapy for Ovarian Cancer*: A Systematic Review

**DOI:** 10.3389/fonc.2021.703949

**Published:** 2021-07-08

**Authors:** Yue Han, Lili Jiang, Kuiran Liu, Ling Ouyang, Yan Li

**Affiliations:** Department of Obstetrics and Gynecology, Shengjing Hospital of China Medical University, Shenyang, China

**Keywords:** ovarian cancer, HE4 – human epididymis protein 4, platinum-resistant, chemotherapy, meta-analysis

## Abstract

**Objective:**

To evaluate the value of serum Human epididymis protein 4 (HE4) for predicting the resistance of ovarian cancer (OS) to platinum chemotherapy.

**Method:**

We searched the MEDLINE (PubMed), EMBASE, Cochrane Central, Web of Science, SCOPUS, and CNKI databases and screened all studies evaluating serum HE4 for predicting OC resistance to treatment with platinum. Two researchers independently evaluated the quality of all eligible original studies using QUADAS-2. RevMan 5.4 was used to compile the quality evaluation form. We also performed a meta-analysis with STATA15.1, and Deek’s funnel plots were used to detect any publication bias.

**Results:**

Eight studies were included in the final meta-analysis. Our results showed that the sensitivity and specificity of preoperative serum HE4 in predicting the resistance of OC to platinum chemotherapy was 80% and 67%, respectively. The diagnostic odds ratio was 8, and the AUC was 0.78 (95% CI: 0.75-0.82), whereas the pooled sensitivity and specificity of serum HE4 after the third-cycle of chemotherapies for predicting chemoresistance in OC was 86% and 85%, respectively, with a diagnostic odds ratio of 33 and AUC = 0.92 (95% CI: 0.89 – 0.94).

**Conclusion:**

HE4 may be an effective predictor of platinum-based chemotherapeutic resistance of OC. Serum HE4 levels after the third chemotherapy cycle may be indicative for clinical practice. Further research is needed to validate the significance of HE4 in the long-term management of OC.

**Systematic Review Registration:**

https://www.crd.york.ac.uk/prospero/, PROSPERO (CRD42021220099).

## Introduction

As the leading cause of gynecologic tumor-related mortality worldwide, ovarian cancer (OC) proves fatal for 18000 of the 290000 women annually diagnosed with the disease (1). Due to its lack of typical clinical symptoms, 80% of the patients present with advanced stages of the disease at the time of first diagnosis (2). Currently, primary debulking surgery combined with platinum-based chemotherapy is the standard treatment for advanced OC. However, 80% of patients with advanced OC eventually relapse and develop resistance to treatment with platinum, which is unfortunately characteristic of a poor prognosis (3). Patients with disease progression during first-line treatment or within six months of the end of chemotherapy completion are considered platinum-resistant (4), requiring second-line chemotherapy. In this group of patients this problem causes a considerable delay in the potential use of more effective therapies (5). Despite the progress in diagnosis and treatment of these patients, in advanced OC the five-year survival rate remains at 30%.

Therefore, finding novel markers is of great clinical significance to provide accurate identification of OC patients that are platinum-resistant. These OC patients could then be switched sooner to a more effective therapeutic regimen that would extend their overall survival. Previous studies have shown that the size of residual lesions and chemotherapy response are the most critical indicators that affect the prognosis of patients with OC. Despite this, there is currently no effective indicator to predict the patient’s response to chemotherapy. While studies have shown CA125’s ability to predict the recurrence of OC (6), there is currently no conclusive result that can be used to predict sensitivity to chemotherapy.

Human epididymis protein 4 (HE4) is mainly expressed in the reproductive and respiratory tracts and is overexpressed in OC (7, 8). Recently, HE4 has drawn attention as a promising marker in the early detection and prognosis of OC (9, 10). Some studies indicate a potential predictive effect of HE4 in the response of OC to chemotherapy. However, according to these studies these results remains controversial.

To date, there have been no studies that comprehensively explain the applicability of HE4 to the response of OC to platinum-based chemotherapy. Therefore, this research aims to systematically review all eligible published studies to determine whether the HE4 might serve as a biomarker of the response to treatment of patients with OC and treated with platinum-based chemotherapy.

## Materials and Methods

### Search Strategy

Two reviewers were assigned primary responsibility for the literature search in the MEDLINE (PubMed), EMBASE, Cochrane Central, Web of Science, SCOPUS, and CNKI databases between January 1949 and February 2021. In addition, each reviewer re-assessed the relevance of the studies found for inclusion in the present study. We used the terms “ovarian neoplasms”[MeSH Terms] OR “ovarian neoplasms” [All Fields] OR “ovary cancer” [All Fields]) OR “OCs” [All Fields]) OR “cancer of ovary” [All Fields]) OR “cancer of the ovary” [All Fields]) OR “ovary neoplasm” [All Fields]) OR “ovarian tumor.” These previously mentioned terms were combined with AND (“human epididymis secretory protein 4” [All Fields] OR “human epididymis protein 4” [All Fields]) OR “HE4” [All Fields]) OR “WFDC2” [All Fields]. The reference lists of all primary studies that qualified for inclusion in our study were reviewed to identify any additional relevant studies for inclusion in the present study.Our registration number is: CRD42021220099.

### Study Selection and Eligibility Criteria

**Figure 1:**
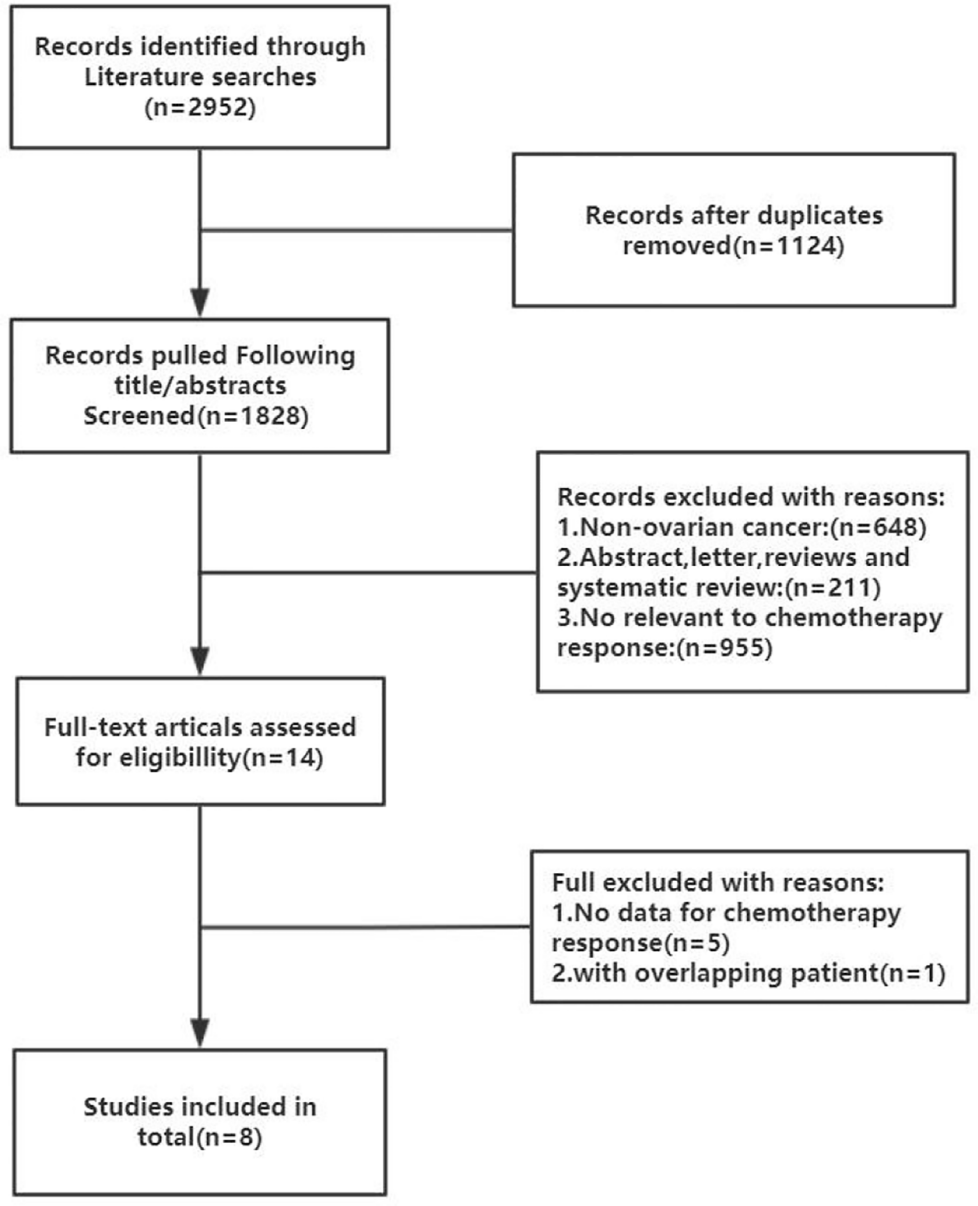
Flow chart of the literature search and selection process in the MEDLINE (PubMed), EMBASE, Cochrane Central, Web of Science, SCOPUS, and CNKI.

The inclusion criteria comprised of the following conditions: (1) Histologically confirmed OC; (2) treatment with a platinum-based chemotherapy regimen; (3) evaluation of the association between HE4 and chemotherapy outcome; and (4) published in English or Chinese. The exclusion criteria included: (1) an inability to access the full text of a study. According to these criteria, a total of eight studies were selected. **Figure 1** is a flow chart of the process used to select studies that were eligible for inclusion in our study.

### Quality Assessment

In our evaluation, we utilized the software Quality Assessment of the Diagnostic Accuracy Studies 2 (QUADAS-2) and RevMan 5.4. STATA15.1 software was used to perform quality assessments of the eligible studies (11). The QUADAS-2 contained 14 items, with each key domain containing two sections: risk of bias, and applicability. We also considered the risk of bias to be low if the answer to all signaling questions within a domain was ‘yes.’ If any answer was ‘no,’ it indicated that potential bias was possible. Concerns about applicability were judged as ‘low,’ ‘high,’ or ‘unclear.’

### Statistical Analysis

All statistical analyses were implemented using STATA15.1. The I2 test and Q test software were used to assess the heterogeneity of the study, and an I2 > 50% was used as an indication of the presence of heterogeneity. We used bivariate regression models to calculate pooled sensitivity; specificity; positive and negative likelihood ratios (PLRs and NLRs); diagnostic odds ratio (DOR); and their respective 95% confidence intervals (CIs). The diagnostic value of the experiment was reflected by calculating the area under the summary receiver operator characteristic curve (SROC, AUC). An age of 50 years and the cut-off value of HE4 were used for subgrouping the patients for analysis, and publication bias was assessed using Deek’s funnel plots.

## Results

### Characteristics of Eligible Literatures

**Figure 2:**
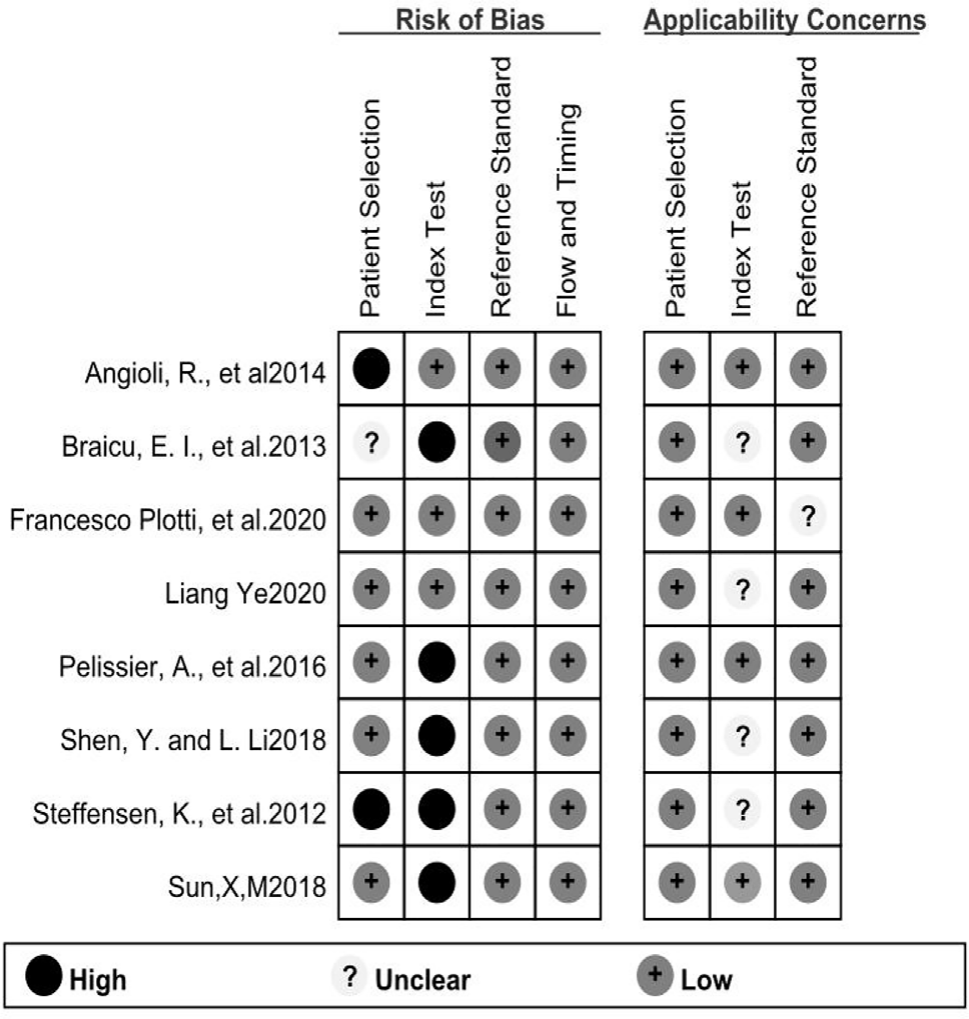
The tabular presentation of QUADS-2 results.

**Figure 3:**
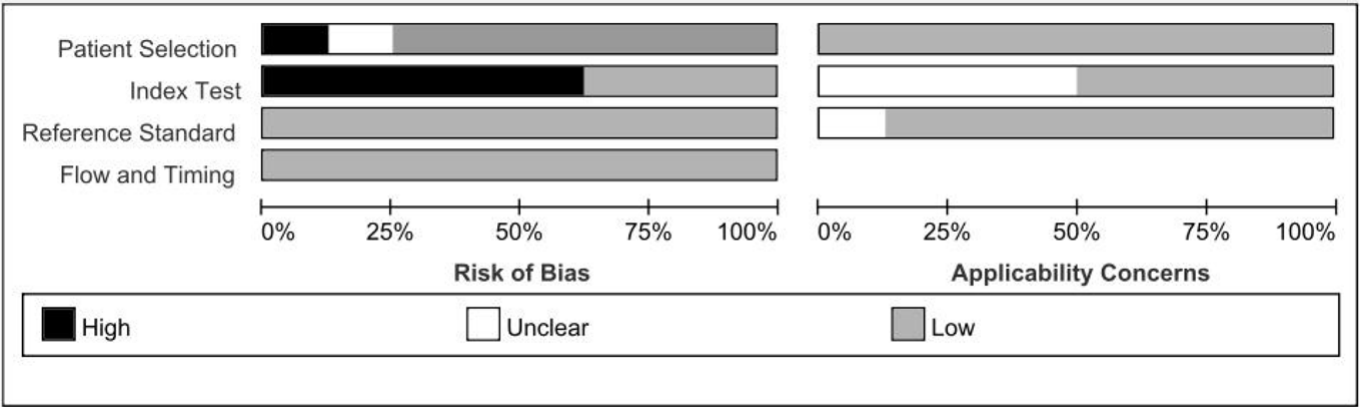
The graphical of QUADAS-2 results.

A flow chart of the literature screening process is shown in **Figure 1**. Initially, 2952 articles were retrieved from the databases, and 1124 duplicates were removed. Then, we excluded 648 articles unrelated to OC, and 955 articles did not evaluate the relationship between HE4 and the response to treatment wityh chemotherapy; subsequently, 211 abstracts, letters, reviews, and systematic reviews were excluded from our study. We then retrieved the full text of 14 articles for further evaluation of their eligibility for inclusion in our study. The results showed that there were five studies from which the outcome data could not be extracted. The studies documented in two articles were based on almost the same set of patients. Eventually, we quantitatively analyzed the eight articles that met the requirements for inclusion in our study (12–19). The relevant characteristics of these eight articles are summarized in **Table 1**. In our study we analyzed eight studies published from 2012 to 2020, and involved a total of 705 patients with OC. The results of the quality evaluation diagram are shown in **Figures 2**, **3**. All eight eligible studies obtained moderately high scores in the QUADAS-2 quality assessment, which indicated that the meta-analysis would be credibility. Retrospective studies often have some bias with regards to the selection of patients. Using a certain cut-off value of HE4 also affected the index test, but these effects on the results of this study were not significant.

**Table 1:** The general characteristics of the 8 included studies.

Author	Year	Area	Meanage	Detection time	Sample size	Cutoff	TP	FP	FN	TN	Method	Histological
Angioli, R., et al. (12)	2014	Italy	50	prechemotherapy	42	70	36	14	0	26	EIA	E0C
Pelissier, A., et al. (14)	2016	French	62.7	prechemotherapy	30	115	11	5	1	13	ECLIA	E0C
Braicu, E. I., et al. (20)	2013	Germany	58	prechemotherapy	275	250	49	109	20	97	EIA	0C
Braicu, E. I., et al. (20)	2013	Germany	58	prechemotherapy	275	400	40	84	29	122	EIA	0C
Steffensen, K., et al. (15)	2012	Danish	64	prechemotherapy	137	502	45	34	14	44	ELISA	E0C
Sun, X, M (16)	2018	china	52.5	prechemotherapy	31	715.7	9	1	4	17	ECLIA	E0C
Shen, Y. and L. Li (17)	2016	china	49.2	prechemotherapy	52	495.04	A	6	5	25	ECLIA	E0C
Angioli, R., et al. (12)	2014	Italy	50	After the third chemotherapy	42	70	36	6	0	34	EIA	E0C
Sun , X, M (16)	2018	china	52.5	After the third chemotherapy	31	77.13	10	3	3	15	ECLIA	E0C
Shen, Y. and L. Li (17)	2016	china	49.2	After the third chemotherapy	52	127.4	10	3	3	15	ECLIA	E0C
Liang Ye (18)	2020	china	54.3	After the third chemotherapy	69	66.58	16	18	4	31	EIA	0C
Francesco Plotti et al. (13)	2021	Switzerland.	61	After the third chemotherapy	69	70	26	1	7	35	EIA	E0C

### Heterogeneity Test

The STATA15.1 software was used for testing the heterogeneity. The results showed that the I2 of preoperative serum HE4 was 74 when predicting OC resistance to platinum chemotherapy and that the heterogeneity due to the threshold effect was 0.04. Similarly, the I2 of serum HE4 after the third round of chemotherapy in predicting OC resistance to platinum was 49, and the heterogeneity caused by the threshold effect was 0. Since each original study and the merge did not fall on the same line in the DOR Forest plot, we synthesized the statistics from all of the original studies.

### Meta Analysis

**Figure 4:**
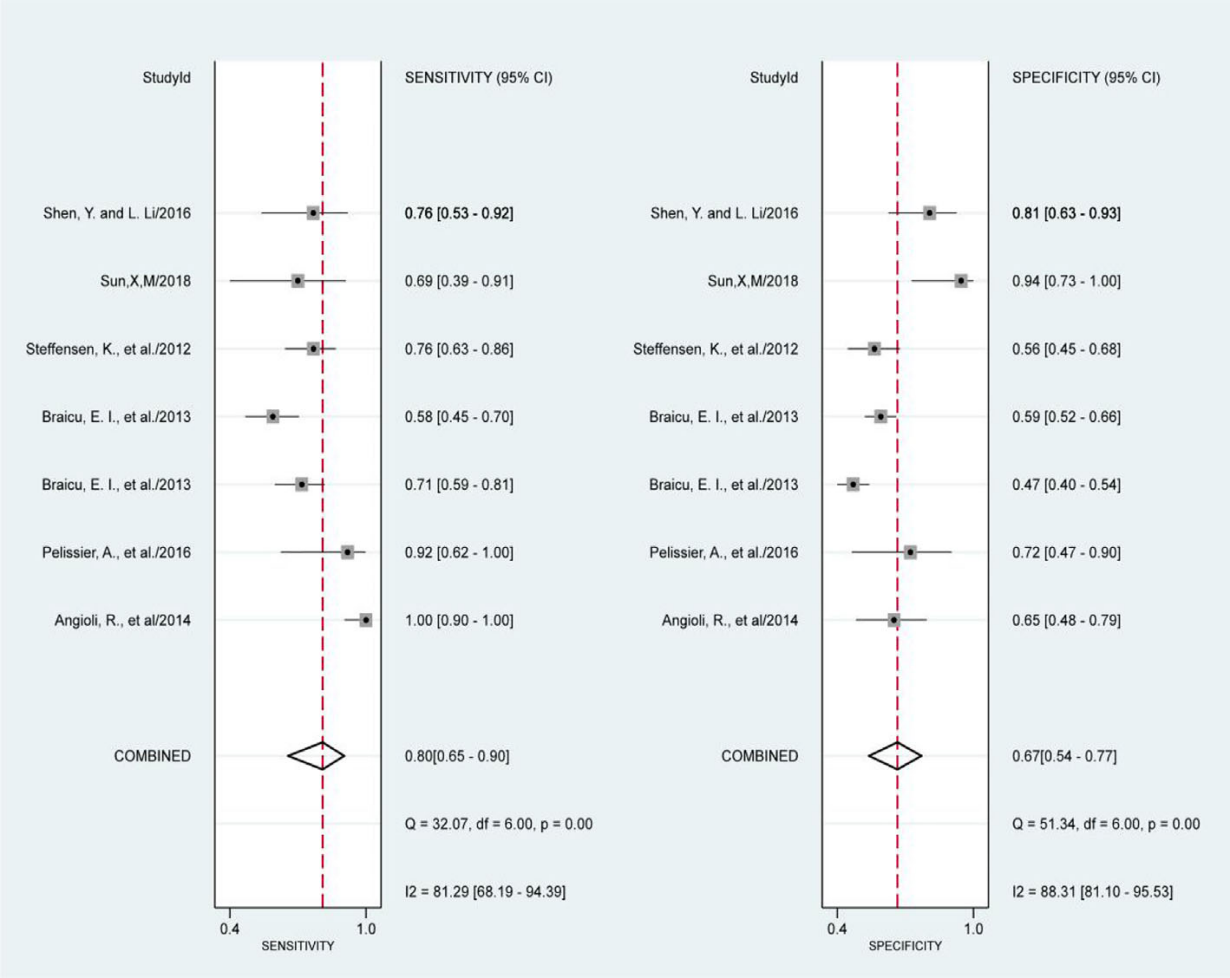
Forest Plots of paired sensitivity and specificity for HE4 after third chemotherapy.

**Figure 5:**
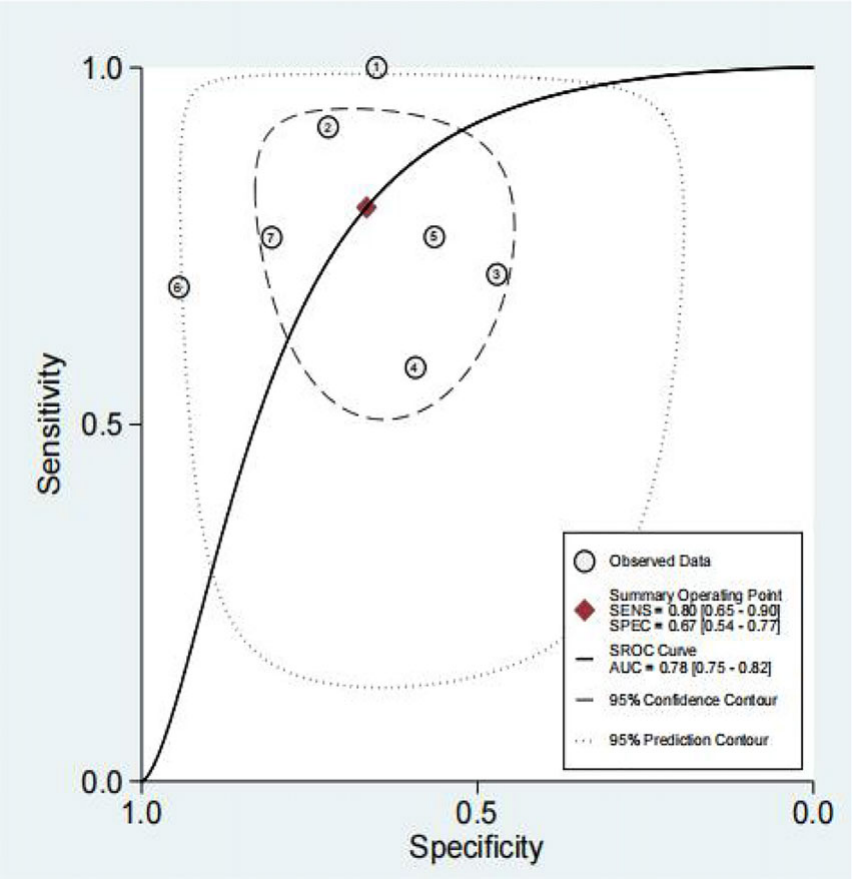
The SROC curve of preoperative serum HE4.

The results of the preoperative serum HE4 meta-analysis at predicting the resistance of OC to platinum chemotherapy are summarized in **Table 2**. The results suggest that the combined sensitivity and specificity were 80% and 67%, respectively, while the diagnostic odds ratio was 8. Some sensitivity and specificity forest plots are provided in **Figure 4**. The SROC curve is shown in **Figure 5** and has an AUC = 0.78 (95%CIs:0.75-0.82).

**Table 2:** The combined predictive value of preoperative serum HE4 in 8 included studies.

Index	Merge value	95%CIs	I2(%)	Cochran–Q	P
Sen	0.80	0.65–0.90	81.29	32.07	0.00
Spe	0.67	0.54–0.77	88.31	51.34	0.00
DOR	8.00	3.00–22.00	87.26	47.11	0.00
PLR	2.40	1.60–3.60	82.04	53.76	0.00
NLR	0.29	0.15–0.58	86.13	43.26	0.00

**Figure 6:**
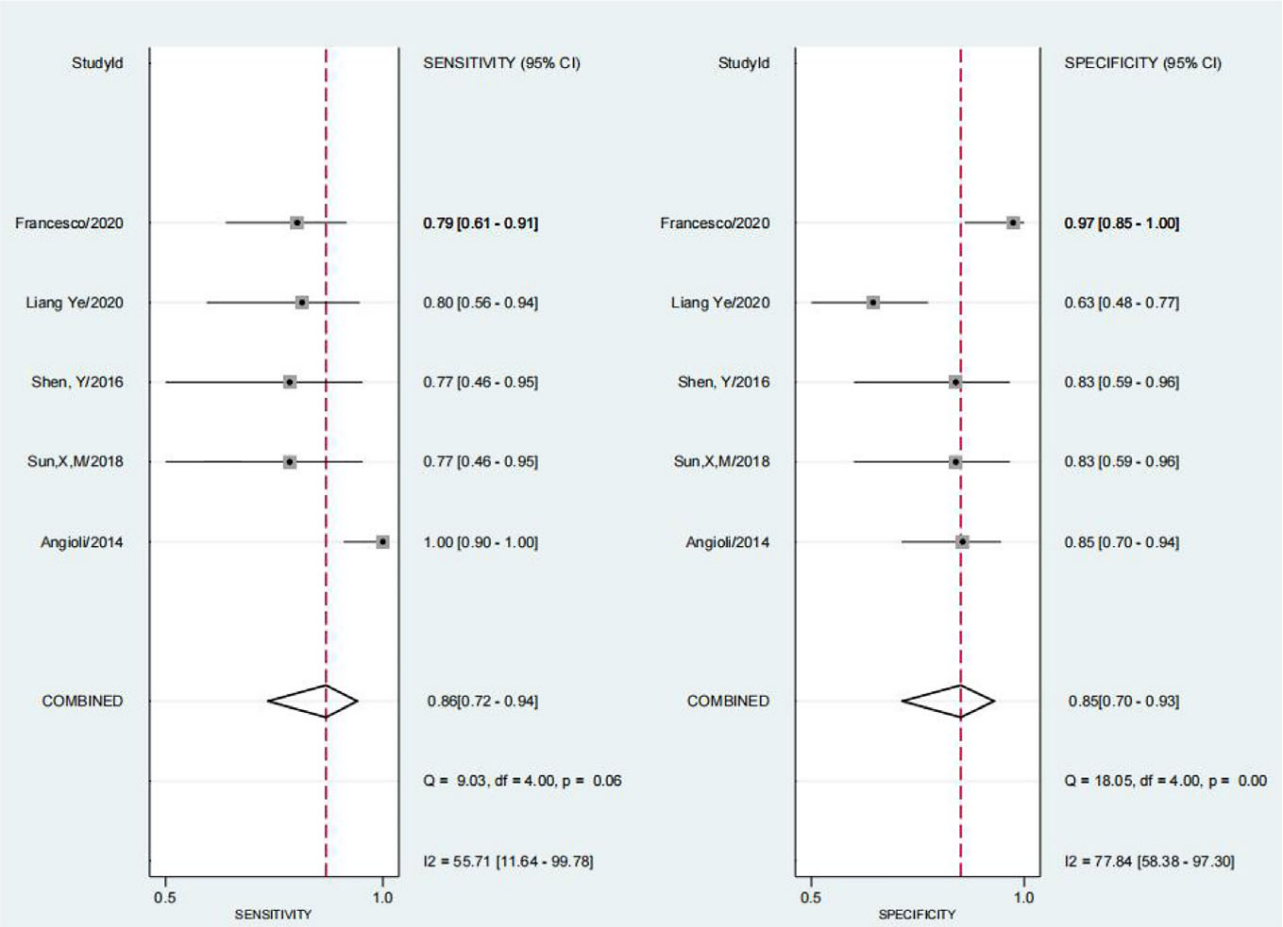
Forest Plots of paired sensitivity and specificity for preoperative serum HE4.

**Figure 7:**
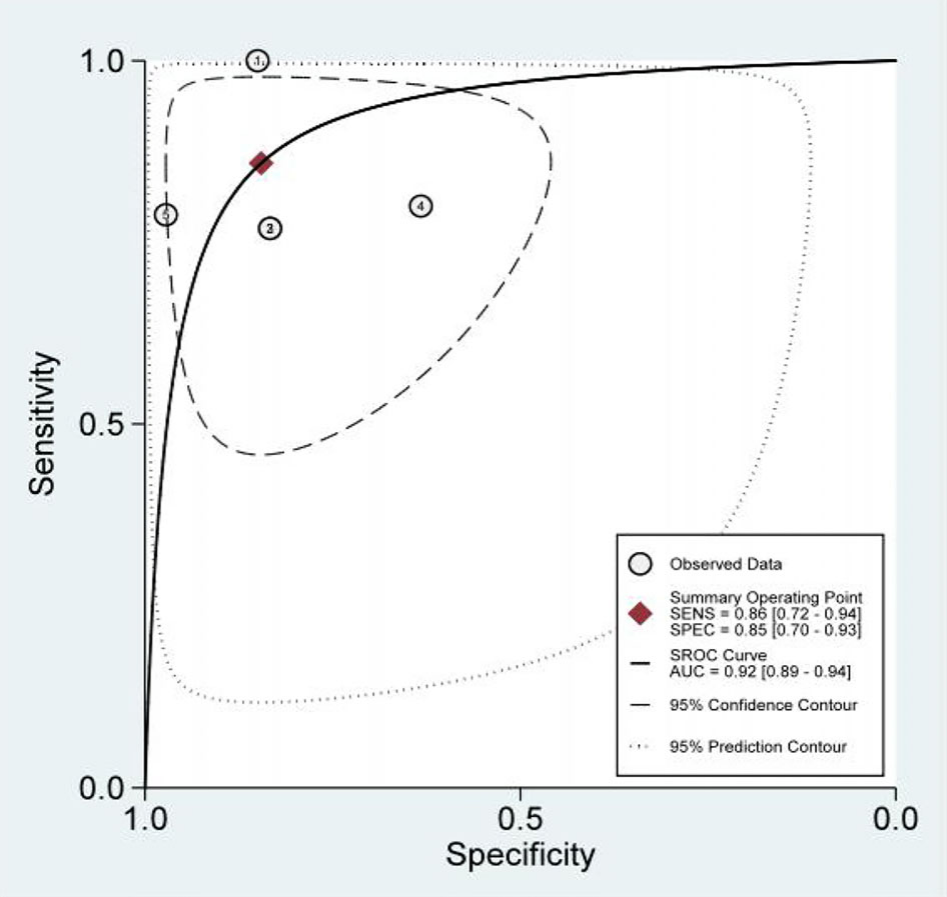
The SROC curve of serum HE4 after third chemotherapy.

We obtained a pretest probability of 0.3 and a posttest probability of 0.51 by plotting the Fagan plot. The details of serum HE4 and the meta-analysis after the third round of chemotherapy, at predicting chemoresistance by OC are shown in **Table 3**. The pooled sensitivity and specificity was 86% and 85%, respectively, and the diagnostic odds ratio was 33. Some Forest plots for sensitivity and specificity are presented in **Figure 6**. The SROC curve is shown in **Figure 7** and has an AUC = 0.92 (95%CIs:0.89-0.94). The Fagan plot indicates a pretest probability of 0.42 and a posttest probability of 0.80. The above results suggest that serum HE4 after the third round of chemotherapy, may have a higher predictive value for OC resistance to platinum chemotherapy.

**Table 3:** The combined predictive value of serum HE4 after third chemotherapy in 5 included studies.

Index	Merge value	95%CIs	I2 (%)	Cochran–Q	P
Sen	0.86	0.72–0.94	55.71	9.03	0.06
Spe	0.85	0.70–0.93	77.84	18.05	0.00
DOR	33.00	10–122	61.45	10.38	0.03
PLR	5.50	2.7–11.4	55.56	17.01	0.00
NLR	0.17	0.08–0.36	32.77	5.95	0.20

We performed a meta-regression analysis on the eight studies based on the cut-off value of HE4 and the mean age. Finally, in the exploration of the predictive value of preoperative HE4 (**Table 4**), the P values were greater than 0.05 with the exception of the P-value for sensitivity of the cut-off subgroup, which was equal to 0.05, while the P-value of the cut-off subgroup was < 0.001 in the joint model. Similarly, in the meta-analysis of the predictive value of serum HE4 after the third round of chemotherapy (**Table 5**), the P-value of the cut-off value was less than 0.05 in the joint model analysis only. The P value of the remaining subgroups were greater than 0.05, indicating that the difference in the cut-off value may have caused heterogeneity in this study.

**Table 4:** meta-regression of preoperative HE4.

Parameter	Category	No. of studies	Sensitivity	Specificity	P
meanage> 50	Yes	6	0.81 (0.68–0.94)	0.62 (0.52–0.73)	0.38
	No	1	0.77 (0.42–1.00)	0.81 (0.63–0.99)	
cutoff> 140	Yes	5	0.69 (0.61–0.78)	0.66 (0.52–0.80)	0.00
	No	2	0.98 (0.94–1.00)	0.69 (0.47–0.90)	

**Table 5:** meta-regression of HE4 after third chemotherapy.

Parameter	Category	No. of studies	Sensitivity	Specificity	P
meanage> 50	Yes	4	0.87 (0.77–0.98)	0.85 (0.73–0.97)	0.84
	No	1	0.78 (0.45–1.00)	0.84 (0.59–1.00)	
cutoff> 140	Yes	4	0.87 (0.77–0.98)	0.89 (0.83–0.95)	0.04
	No	1	0.81 (0.53–1.00)	0.63 (0.47–0.79)	

### Sensitivity Analysis and Publication Bias

**Figure 8:**
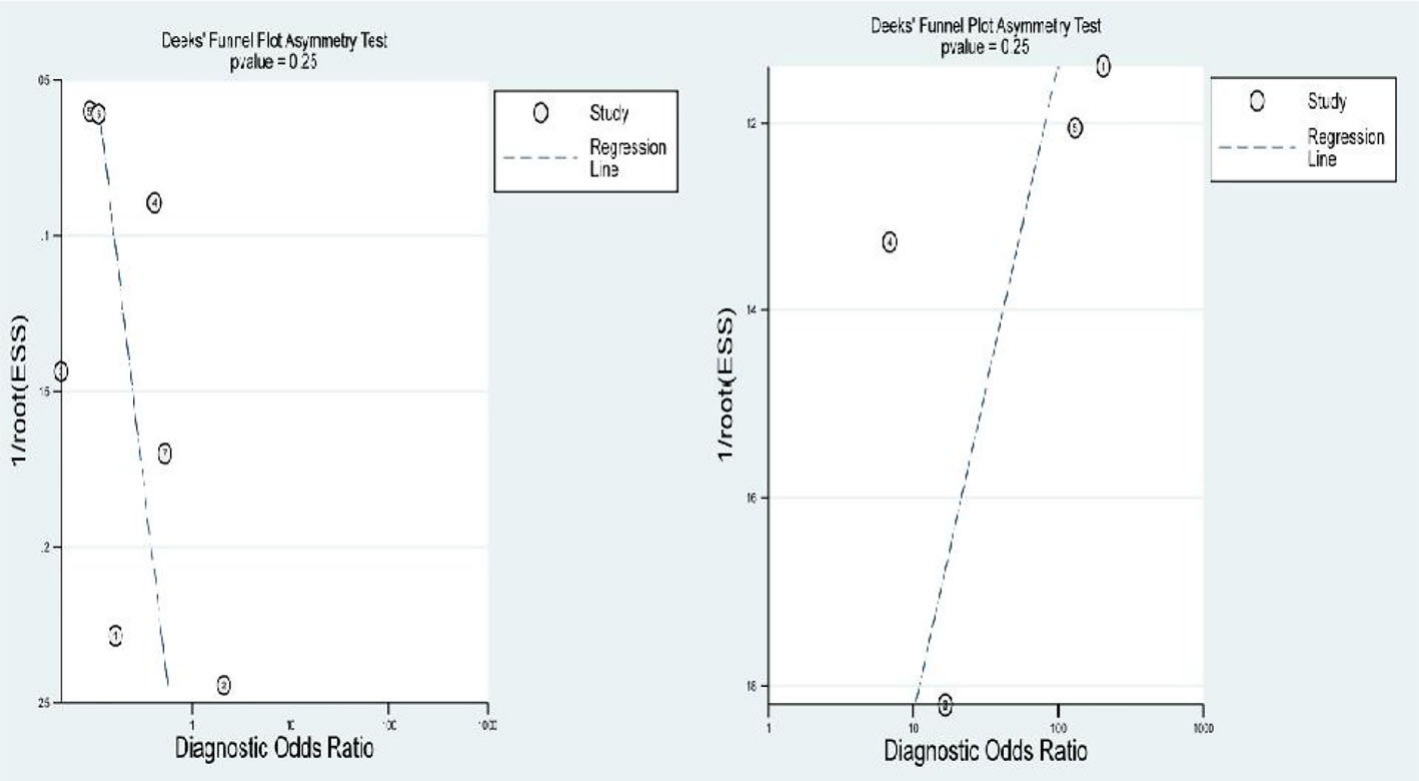
Deek’s funnel plot.

After removing the studies with quite different results, the meta-analysis was performed again, and the results had no visibly effect. This suggests that the results of this study are credible. We detected publication bias by Deek’s funnel plot, and no publication bias was found as shown in the **Figure 8**.

## Discussion

To date, eight studies have investigated HE4’s prognostic value in predicting platinum-based chemosensitivity of OC. However, since the role of HE4 was inconsistent and inconclusive, we reviewed the published articles to evaluate the possibility of the clinical application of HE4 in OC. To the best of our knowledge, this article is the first systematic review that discusses the relationship between HE4 and platinum-based chemotherapy sensitivity in OC. Previous studies have demonstrated HE4’s important role in the diagnosis and prediction of the prognosis of OC and this has been consistently proven in guiding clinical practice and prolong the survival of patients. In the past decade, breakthroughs have been made in surgical methods for treating OC and further drug research, and the 5-year survival rate has improved.

However, platinum chemotherapy resistance is still a complex problem to overcome in the long-term management of OC. Although CA125 may be effective in monitoring the response of OC to treatement, its sensitivity and specificity require substantial improvement. This therefore, underscores the practical significance of exploring other more effective serological markers. Currently, HE4 is considered a promising tumor marker. Previous studies have suggested that HE4 levels are elevated earlier than CA125 levels as the disease progresses (21, 22).

Overall, this systematic review and meta-analysis contains eight eligible studies involving 705 OC patients, and the data from these studies are summarized in **Table 1**. Thus far, a variety of studies have revealed that HE4 expression is generally different in the platinum-resistant and the platinum-sensitive group. In addition, they indicated that its predicted efficacy is on par with that of CA125 or possibly better. Studies have shown that serum CA125 levels before treatment, at follow-up, and after three rounds of chemotherapy have lower predictive ability for the efficacy of platinum-based chemotherapy on OC than serum HE4 (16–18). Meanwhile, other studies showed no significant difference in CA125 in the resistant control group (12, 19). These results suggest that CA125 may be more advantageous in diagnosing OC, whereas HE4 is a more sensitive predictor of OC resistance to platinum.HE4 promises to be an effective marker for predicting the sensitivity of platinum-based chemotherapy for OC, providing a direction for future clinical research. In this study, we used meta-analysis to examine the value of serum HE4 in predicting OC resistance to platinum chemotherapy. Given the large difference between preoperative serum HE4 and postoperative serum HE4 expression, we initially explored the predictive value of serum HE4 at the different stages independently.

The results of the heterogeneity test revealed no significant heterogeneity in this study. In fact, the I2 test of serum HE4 after the third round of chemotherapy in predicting OC chemoresistance was 49, and the heterogeneity caused by the threshold effect was 0, and there was no indicated publication bias according to Deek’s funnel plot. These results showed the reliability of this study’s result. After a comprehensive analysis, the pooled sensitivity and specificity were 80% and 67%, respectively, and the positive likelihood ratio was 2.4, the negative likelihood ratio was 0.29, and the AUC was 0.78. It demonstrated that preoperative serum HE4 might be a better indicator for predicting OC resistance to platinum chemotherapy. In comparison, the results of the serum HE4 levels after the third round of chemotherapy were more satisfactory. The pooled sensitivity and specificity were 86% and 85%, respectively, while the positive predictive value was 5.5, the negative predictive value was 0.17, and the AUC was 0.92. The higher sensitivity and specificity are indicative of the relation between HE4 and OC sensitivity to platinum-based chemotherapy.

The abovementioned factors suggest that the predictive significance of HE4 was better after the third round of chemotherapy. Angioli et al. showed that after the third chemotherapy cycle HE4 was closely associated with platinum-based chemotherapy responses (12). This value was considered a strong predictor of the initial outcome of treatment. The researchers used serum HE4 values before the first round of chemotherapy and after the third round of chemotherapy to establish a predictive model. They observed a substantial (47%) decrease in HE4 between the first and third rounds of chemotherapy. They used this as a cut-off value to risk-stratify patients and divided them into platinum-resistant high-risk or low-risk groups. Since the HE4 value after the third chemotherapy cycle could be used to screen patients with platinum-based chemotherapeutic resistance, it is possible to prolong patient survival time for those receiving second-line chemotherapy drugs, earlier.

Additionally, Angioli (12) and Sun et al. (23) indicated that after the third chemotherapy cycle, that serum HE4 is closely related to OC resistance to platinum chemotherapy. Cannistra et al.’s study (24) and the AURELIA test (25) suggest that single drug or combined bevacizumab chemotherapy has a particular effect on patients with platinum-resistant OC. Furthermore, Yosuke Tarumi (26) attained long-term survival after 32 cycles of bevacizumab and 30 months of observation in the treatment of platinum-resistant OC and without disease progression. As the primary ovarian maintenance treatment process, PARP inhibitors are primarily targeted therapies in platinum resistance OC with improvement to survival (27–29). The eight studies included in the present study, suggested the role of HE4 in predicting chemoresistance in OC and emphasized the significance of serum HE4 after the third cycle of chemotherapy to predicting chemosensitivity. If further research provides further support that third cycles of chemotherapy after serum HE4 expression can predict OC platinum resistance, these patients can preemptively choose second-line chemotherapy drugs or targeted drugs nine months in advance, which provides new hope for patients with OC.

Studies have shown that HE4 is highly expressed in gynecological tumors and pancreatic cancer. Li et al. (30) reported that HE4 overexpression in endometrial cancer cells promoted cell proliferation, stromal infiltration, and other malignant behaviors of cancer cells. HE4 overexpression promotes the proliferation of CAPAN-1 pancreatic cells and significantly reduces the cells’ paclitaxel sensitivity (31). Ribeiro et al. (32) indicated that overexpression of HE4 resulted in increased resistance of the SKOV3 and OVCAR8 OC cell lines to cisplatin and paclitaxel, and that HE4 knockout could partially reverse resistance. Similarly, Lee’s (33) study confirmed that He4-overexpressing cells activate the AKT and ERK pathways through the growth signaling pathway of the epidermal growth factor cell, which resulted in a dose-dependent decrease in cisplatin activity. In an OC mouse model, HE4 overexpression promoted the growth of a transplanted tumor and the chemotherapeutic cisplatin resistance (4).

Currently, there are many methods for detecting serum HE4. However, due to different detection principles of various detection methods, the accuracy of detection results also differs slightly. HE4 EIA is a method for the quantitative determination of serum HE4 content by enzyme-linked immunosorbent assay with a detection range of 15-900 pmol/l. Specimens may be held at 2-8°C for up to 3 days before testing, and the total coefficient of variation (CV) of the precision determined by HE4 EIA is <15%. Electrochemiluminescence immunoassay (ECLIA) has a total CV of detection precision of <5%. It combines luminescence technology, the immune response, and computer technology, with the advantages of high detection accuracy and a high degree of automation. While the link enzyme-immunosorbent assay (ELISA) has the advantages of easy operation and low cost, it frequently causes result deviation and produces lower stability and repeatability than ECLIA. Most of the studies included in this review used ECLIA with high precision, while only one study used ELISA.

Age is an important factor that affects serum HE4 levels. There are conspicuous differences in serum HE4 levels among different age groups, which gradually increases with age and significantly increases after 60 years of age. When comparing postmenopausal and premenopausal women, researchers found that serum HE4 levels were significantly increased in postmenopausal women. However, further comparison of premenopausal women aged 40 years and older and postmenopausal women under 60 years revealed no statistically significant difference in serum HE4 levels (34). This suggested that age may be more important for the effect of HE4 levels. However, Cheng et al. (35) concluded that both age and menopausal status are important factors that affect HE4 levels. The difference in the conclusions between the two studies may be because they used different definitions of menopausal status. The former defined age under 45 as premenopausal women and lacked samples in the age range of 46 to 54 years, while the latter described more than one year of amenorrhea as the menopausal status. A Korean study involving 1809 healthy people showed that HE4 levels gradually increased in people over 50 years of age; these levels seemed to be influenced by age rather than menopausal status (36). Conversely, Tian demonstrated that age was not an independent factor using a multivariate analysis (37). The populations involved in this systematic review were around the age of 50 years, the largest mean age was 64 years, and the youngest was 48 years, with possible age-related differences. In this study, the age of 50 was used as the cut-off value for analysis. The results showed that the effect of age on the heterogeneity of the results was not statistically significant. This is likely due to the small sample size of the analysis and should be confirmed by future studies with larger sample sizes.

Previous studies have shown that HE4 levels generally occur below 140 pmol/l in evidently healthy Western women, while about 98% of all women have HE4 levels below this value. Using the 95th percentile as the cut-off point, Moore analyzed 1101 normal human blood samples and found that the normal cut-off value was 114.8 pmol/l for all women, 89.1 pmol/l for premenopausal women, and 125.6 pmol/l for postmenopausal women (34). Preoperative HE4 or HE4 during chemotherapy can be selected when applying HE4 to predict platinum chemosensitivity in OC. This makes the choice of cut-off value different from the HE4 cut-off value for the diagnosis of OC.

However, there is no clear guideline for specifying the cut-off value of HE4 when predicting chemosensitivity and the definition of the HE4 cut-off value is inconsistent among all included studies, ranging from 70pmol/L to 715.7 pmol/L. In this study, serum HE4 in both the preoperative group and the group after the third cycle of chemotherapy were analyzed at a cut-off value of 140 pmol/l. The preoperative group showed that the heterogeneity of the study results might be derived from the threshold value. However, the threshold in the group after the third chemotherapy cycle did not affect the results. Due to the difference in the cut-off values selected by various original studies, the heterogeneity caused by the threshold effect is large. Therefore, it is vital to establish a standard HE4 cut-off value which may require further research to obtain more meaningful results.

This study has several limitations. Notably, the number of eligible studies included was relatively small. In addition, most of the included studies were retrospective. Larger prospective clinical studies are needed to confirm the significance of HE4 in OC chemoresistance and provide evidence for its application in clinical practice.

## Conclusion

According to a review of previous research, HE4 may be an effective predictor of OC resistance to treatment with platinum-based chemotherapy. After the third cycle of chemotherapy, serum HE4 levels may be indicative in clinical practice. Although the FDA has approved the use of HE4 in the follow-up of OC, there is still a lack of standardized guidelines for clinical application. Considerably more studies are needed to verify the significance of HE4 in the long-term management of OC.

## Data Availability Statement

The original contributions presented in the study are included in the article/supplementary material. Further inquiries can be directed to the corresponding authors.

## Author Contributions

YL: Conceptualization,Methodology,Writing - Review & Editing. YH: literature search, Study selection, Data Curation,Visualization, Writing - Original Draft. LJ: literature search, Study selection, Data Curation. KL: Validation, Writing - Review & Editing. LO: Validation, Writing - Review & Editing. All authors contributed to the article and approved the submitted version.

## Conflict of Interest

The authors declare that the research was conducted in the absence of any commercial or financial relationships that could be construed as a potential conflict of interest.
